# Tetraspanin blockage reduces exosome-mediated HIV-1 entry

**DOI:** 10.1007/s00705-018-3737-6

**Published:** 2018-02-10

**Authors:** Brian Sims, Anitra L. Farrow, Sparkle D. Williams, Anju Bansal, Alexandre Krendelchtchikov, Qiana L. Matthews

**Affiliations:** 1 Division of Neonatology, Departments of Pediatrics, Neurobiology and Cell, Developmental and Integrative Biology, University of Alabama at Birmingham, Birmingham, USA; 2 Center for AIDS Research, University of Alabama at Birmingham, Birmingham, USA; 3 Division of Infectious Diseases, Department of Medicine, University of Alabama at Birmingham, Birmingham, USA; 4 Microbiology Program, Department of Biological Sciences, College of Science, Technology, Engineering and Mathematics, Alabama State University, Montgomery, AL 36104 USA

## Abstract

HIV-1 is one of the most studied retroviruses. The role of exosomes in HIV-1 entry and pathogenesis are beginning to be appreciated. Exosomes can incorporate host proteins that are also contained in viruses (e.g., tetraspanins).

## Introduction

Human immunodeficiency virus-1 (HIV-1) is one of the most studied retroviruses; however, there is still a wealth of information to be learned about this virus’s entry, lifecycle and pathogenesis. The role of exosomes in HIV-1 entry and pathogenesis is beginning to be appreciated. Exosomes are small extracellular vehicles, which traffic nucleic acids (RNA, microRNA, and DNA), proteins, and lipids. Exosomes are secreted by most cell types into the extracellular milieu and then internalized into the recipient cells. It has been well documented that exosomal composition reflects the donor cells in which they are generated. Exosomes can incorporate host proteins that are also contained in viruses (e.g., tetraspanins) [1, 2]. Furthermore, exosomes appear to affect viral infection [3–9]. It has been previously demonstrated that blocking cluster of differentiation (CD) 9 and CD81 tetraspanins, which are commonly expressed on exosomes, resulted in significant decreases in the exosomal uptake efficiency in dendritic cells [10]. Regarding HIV-1, the role of exosomes in pathogenesis is complex. Studies have shown that exosomes may promote or inhibit HIV-1 infection [11, 12]. The overlap between HIV-1 and exosome biogenesis within an infected cell suggests that HIV-1 products, including RNA and proteins may be encased within exosomes or contaminate exosome preparations from HIV-1 infected fluids [13–15].

We are just beginning to unravel the complex nature of exosomes and HIV-1 interactions via lipids, phospholipids and proteins [16, 17]. Our group has recently shown that HIV-1 entry into human immune cells is enhanced by exosome-mediated trafficking [9]. This effect was illustrated with exosomes derived from human plasma, human breast milk, mouse neural stem cells and human lung carcinoma cells. Furthermore, our study demonstrated that HIV-1 and exosome interactions were mediated partially through binding of the T cell immunoglobulin and mucin protein 4 (TIM4) to the viral envelope [9]. In this study, we demonstrated that exosomes can enhance HIV-1 entry into human T and monocytic cell lines via exosomal tetraspanin proteins CD81 and CD9.

## Materials and Methods

### Cell culture

Human CD4^+^ lymphoblastoid T cell line (line A3R5.7) was a gift from the UAB CFAR Virology core. These cells were commercially received from the NIH AIDS Research and Reference Reagent Program and subsequently genetically modified. A3R5.7 cells were maintained in RPMI 1640 medium supplemented with 10% heat-inactivated, exosome-free fetal bovine serum, 2 mM l-glutamine, penicillin (100 U/mL), streptomycin (100 μg/mL) (Thermo Fisher Scientific, Waltham, MA, USA), and 1 mg/mL geneticin (G418; Thermo Fisher Scientific). Human monocytic cells (line THP2574) were maintained in similar medium but without geneticin [18, 19]. All other cell lines were purchased from American Type Culture Collection.

### Exosome purification

#### Isolation of human embryonic kidney cells (HEK 293)-derived exosomes

Cell line HEK293 was grown in DMEM-F12 complete medium containing exosome-free fetal bovine serum to ~80% confluency. In brief, cells were centrifuged at 5,000 rpm for 10 min at 4°C using a Sorvall RT600 centrifuge with a swinging bucket rotor (Thermo Fisher Scientific). The supernatant was clarified by filtration through a 0.22 μm filter and centrifuged at 13,200 rpm for 70 min at 4°C using an SW41T1 swinging rotor in a Beckman Coulter (Brea, CA, USA) Optima L-70K ultracentrifuge for exosome collection [8, 9, 20]. Exosomes were resuspended in 120–450 µL sterile phosphate-buffered saline (PBS) and then quantified by Bradford protein quantitation method.

### Isolation of breast milk-derived exosomes

Breast milk samples were retrieved from remnants of breast milk samples from human donors and centrifuged twice at 3,500 rpm for 10 min at 4°C. The fat layer was aspirated and the supernatant transferred to a new tube. A third spin was performed at 5,000 rpm for 30 min at 4°C, after which the remaining fat was aspirated and the supernatant transferred to a new tube. Breast milk was then filtered with a 0.22 µm filter, transferred into an ultracentrifuge tube and then the tube volume was adjusted with PBS prior to an ultracentrifugation spin at 32,000 rpm for 70 min at 4°C. The pellet was collected and resuspended in 120–450 µL sterile PBS.

### Isolation of human plasma-derived exosomes

Plasma was collected from whole blood of human donors into tubes containing ethylenediaminetetraacetic acid (EDTA) and processed as described by Konadu et al [21] with some modifications. Whole-blood samples were centrifuged at 3,500 rpm for 10 min at 4°C. If the samples contained a high lipid content after the low-speed centrifugation (evidenced by color), they were incubated for 2 h at 4°C, and the precipitated fat was removed by centrifugation at 5,000 rpm for 10 min at 4°C. The supernatants were then filtered through a 0.22 μm filter and ultracentrifuged for 30 min at 13,200 rpm in a SW41T1 swinging rotor at 4°C. The pellet was collected by centrifug-ing the samples at 27,000 rpm for 2 h at 4°C. The resulting pellet was resuspended in 1 mL PBS, loaded on to OptiPrep velocity gradients, and subjected to flotation centrifugation at 27,000 rpm for 2 h. Fractions with peak exosome content (fractions 2 and 3) were pooled, diluted with PBS, and ultra-centrifuged for 2 h at 32,000 rpm. The exosome pellet was resuspended in 120 µL PBS.

### SDS-PAGE and Western blot analyses of exosome-associated proteins

To analyze proteins associated with exosomes, exosomes were mixed with loading buffer (1:1), boiled, and resolved on a 4%–12% Bis–Tris gel, followed by transfer and blocking on a polyvinylidene difluoride membrane. Blotting was per-formed with two different primary antibodies: anti-clathrin monoclonal antibodies (1:1,000; BD Biosciences, San Jose, CA, USA) and anti-CD81 (1:1,000; System Biosciences, Inc., Palo Alto, CA, USA). Incubation with secondary antibodies was performed using either horseradish peroxidase (HRP)-conjugated goat anti-mouse (1:2,000; Dako Denmark A/S, Glostrup, Denmark) or anti-rabbit (1:20,000; System Biosciences, Inc.) antibodies. Proteins were detected using an enhanced chemiluminescence kit (ELC Western Blotting Substrate Pierce/Thermo Fisher Scientific) and a Bio-Rad ChemiDoc XRS system (Bio-Rad Laboratories, Hercules, CA, USA).

### HIV-1 infection of human cell lines

HIV-1 experiments were conducted using an infectious molecular clone (IMC) (NL-LucR.T2A-YU2.ecto [YU-2]) engi-neered to express *Renilla* luciferase (LucR) [22] and ENV from the YU-2 virus strain, which was derived from the postmor-tem brain of a patient with HIV-associated neurocognitive disorder (HAND)[23]. Throughout this manuscript, this IMC will be referred to as, virus.

A3R5.7 cells were seeded on 96-well plates at a density of 1×10^5^ cells/well with the addition of 5 µg/mL diethylam-inoethyl-dextran [24]. Exosomes derived from NSC, A549, breast milk, and plasma were incubated, respectively, with virus at a multiplicity of infection (MOI) of 0.002 for 1 h at 37°C in 5% CO_2_, [23] and then HIV-1/exosome mixture was co-incubated with cells for 72 h at 37°C in 5% CO_2_. LucR activity was determined using the *Renilla* Luciferase Assay System (Promega Corporation, Fitchburg, WI, USA). Relative luminescence units (RLUs) were measured in triplicate on a Victor X light luminescence counter (PerkinElmer Inc, Waltham, MA, USA) with an exposure time of 0.1 s/well.

THP2574 cells (1×10^4^ cells/well) were classically differentiated into macrophages with 1 ng/mL phorbol 12-myristate 13-acetate for 96 h. The virus with an MOI of 0.18 was incubated with exosomes for 1 h at 37°C in 5% CO_2_. The HIV-1/exosome mixture was co-incubated with the cells for 72 h at 37°C in 5% CO_2_, and LucR activity was measured in triplicate as described earlier.

Amounts of exosomes and viral MOIs used for each cell line were determined experimentally. The following quantities of exosomes, which would saturate all HIV-1 virions, were used for viral entry experiments: 0.035 μg breast milk-derived exosomes, 0.05 μg plasma cell-derived exosomes, and 0.1 μg HEK 293-derived exosomes.

### Blocking of HIV-1 infection

A protocol similar to HIV-1 infection was performed but with addition of 0.2 µg/well anti-human CD9 or CD81 (1:1,000; BD Biosciences, San Jose, CA, USA) to the YU-2/exosome/cell incubation. Virus only was used as control.

### Determining exosome size and concentration

We used nanoparticle tracking analysis (NTA) using the Nano-Sight LM10 (Malvern Instruments, Inc., Malvern, UK) and NTA v2.0 software to characterize breast milk- and plasma-derived exosomes. All data were collected using five frames and in triplicate. Samples were diluted 1:1,000 prior to tracking, which led to particle sizes of 10^−7^–10^−9^ m. Mean values were recorded and analyzed for each given reading frame.

### Human study participants

The study was approved by the Institutional Review Board (IRB) for the Protection of Human Subjects in Research at the University of Alabama at Birmingham (UAB) in accordance with approved guidelines and protocol. Breast milk was collected from samples prior to being discarded at the UAB Regional Newborn Intensive Care Unit with expedited IRB approval. Blood donors provided written informed consent prior to donation.

### Statistical analysis

Data represent 12 independent experiments. Significant differences between treatment groups were determined by one-way ANOVA with a *post hoc* Tukey’s test performed on obtained data points, and results are presented as means ± SEM, *p<0.05, **p<0.01, ***p<0.001, ****p<0.0001.

## Results

### Isolation and characterization of exosomes

**Fig. 1:**
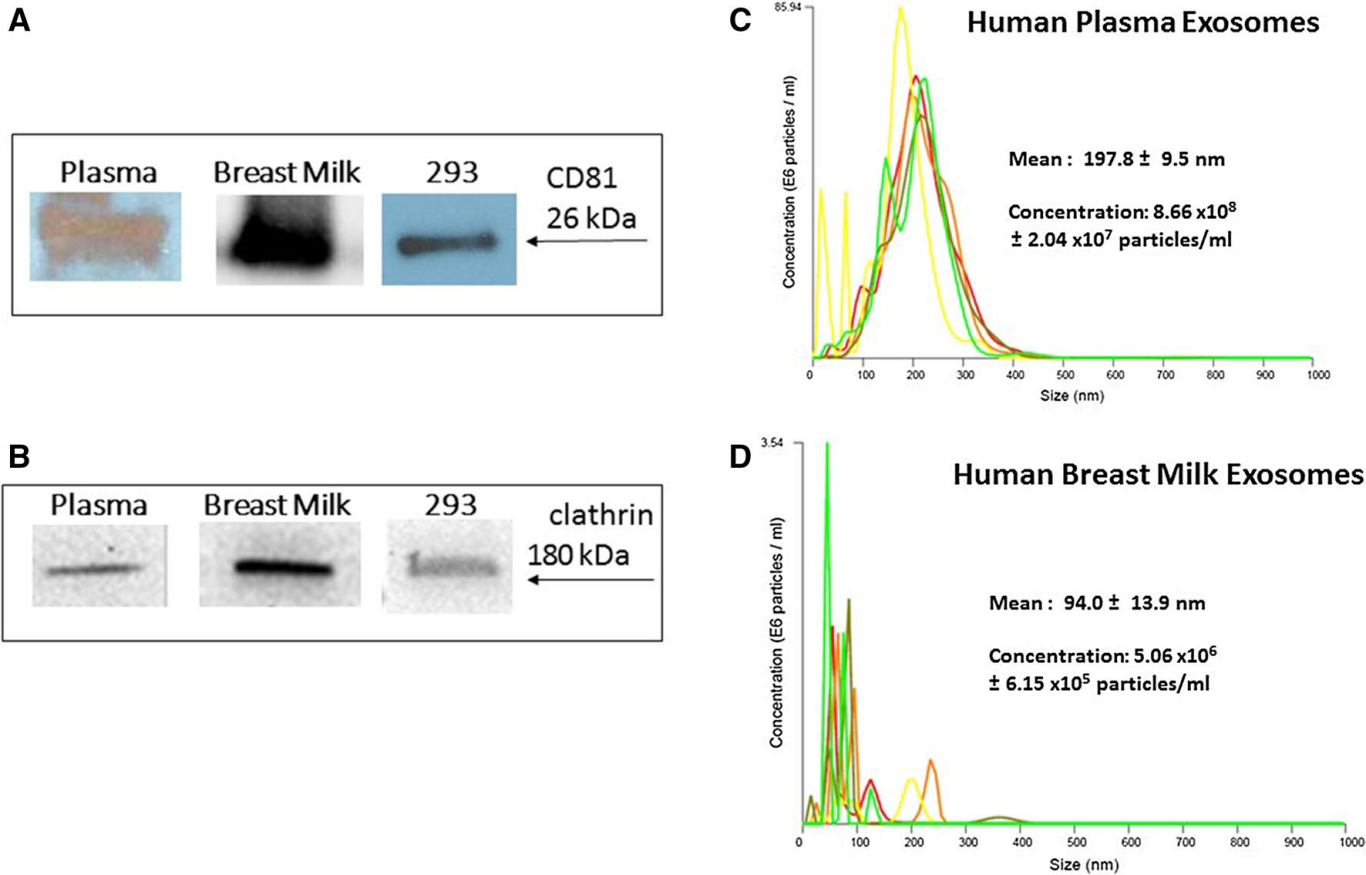
Western blot and nanoparticle tracking analysis (NTA) validation of exosomal samples. Western blots of plasma exosomes, breast milk exosomes, and 293 exosomes. 60 μg/lane, were probed with **(A)** anti-CD81 or anti-clathrin **(B)**. Arrows indicate proteins of interest. NTA-generated size and concentration plots for **(C)** human plasma and **(D)** human breast milk-derived exosomes. The different colors lines represent different pools of exosomes

Exosomes were isolated and characterized for clathrin and exosomal markers, CD81, as described in our previous study [8]. For the current studies, we isolated exosomes from one cell line, HEK 293. We also isolated exosomes derived from human breast milk and human plasma using published methods [8, 9, 21]. To confirm successful exosome isolation, we performed a series of SDS-PAGE and western blot analyses to examine expression of well-known exosomal proteins CD81 (26 kDa) and clathrin (180 kDa) (Fig. 1A and 1B). We used nanoparticle tracking analysis (NTA) as previously performed [9] to validate our exosome preparation from human biological samples (Fig. 1C and D). Human plasma exosomes (Fig. 1C) have a mean diameter size of 197.8 ± 9.5 nm and concentration of 8.66 x10^8^ ± 2.04 x10^7^ particles/ml. Human breast milk-derived exosomes (Fig. 1D) have a mean size of 94.0 ± 13.9 nm.

### Human-derived exosomes augment HIV-1 entry into cell lines and is subsequently blocked by CD81 antibodies

We previously showed that various exosomes augmented HIV-1 entry in human immune cell lines and this entry was partially blocked by TIM-4 blocking [9]. In this current study, we evaluated the impact of tetraspanins on exosome-mediated HIV-1 entry. We used IMC termed NL-LucR.T2A-YU2.ecto, which was also used in our recent work [9]. Throughout this manuscript, this IMC will be referred to as, virus. To determine the impact of exogenous exosomes (human plasma, breast milk, and HEK 293) on HIV-1 entry and subsequent gene expression, we performed a series of viral infection experiments using exosomes only, virus only, exosomes with virus, or exosomes with virus and anti-CD81 antibody. CD81 blocking experiments were based on previously published experimental dose titrations [9]. The above combinations of molecules were added to either the T lymphoblastoid cell line (A3R5.7) or the macrophage-like cell line (THP2574).

**Fig. 2:**
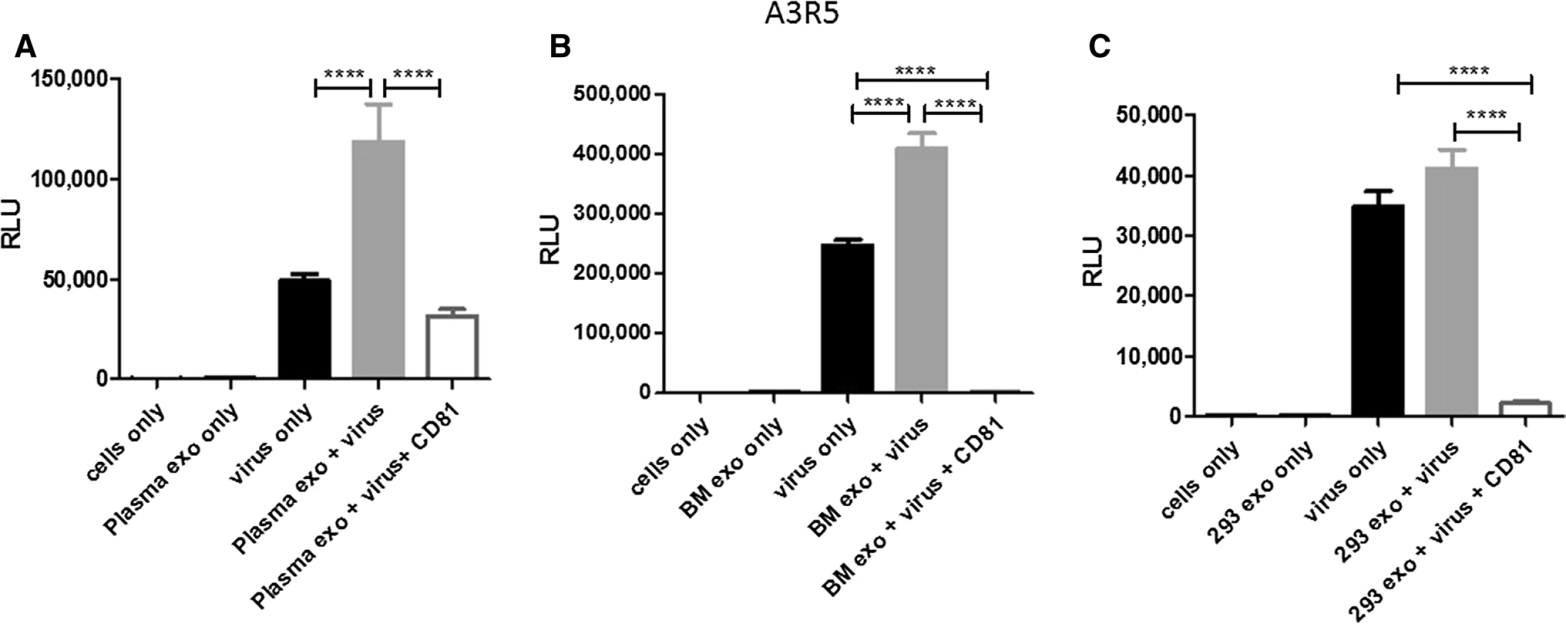
Exosomes significantly enhance HIV-1 entry into a human T cell line. A3R5.7 cells were seeded at a density of 1x10^5^ cells/well with the addition of 5 µg/ml diethylaminoethyl-dextran [9, 24]. Virus was used at a multiplicity of infection (MOI) of 0.002. Virus entry into A3R5.7 cells was evaluated in the presence or absence of **(A)** plasma-derived exosomes (0.05 μg) **(B)** breast milk-derived exosomes (0.035 μg) or **(C)** 293-derived exosomes (0.1 μg). Viral entry was also evaluated in the presence of exosomes and anti-CD81 antibody (0.2 µg/well). All incubations were processed for one hour. Viral gene expression in all control and treatment groups was assessed by *Renilla* luciferase activity at 72 h post-infection. This time point was selected because we previously observed that it allows for optimal expression in a variety of cell types, including those in the present experiments. All conditions are identical through the following experiments unless otherwise noted

Virus alone infected A3R5.7 cells and induced viral gene expression, which was measured at approximately 49,000 relative luminescence units (RLU). Co-incubation of virus and plasma-derived exosomes enhanced infectivity significantly (p<0.0001) compared to virus only. The addition of anti-CD81 antibody significantly (p<0.0001) diminished viral gene expression, illustrating the specificity of CD81/exosome interactions (Fig. 2A). Similar results were observed when the CD81 antibody was used to block exosome-mediated virus entry of breast milk-derived exosomes (p<0.0001; Fig. 2B) and 293 derived-exosomes (p<0.0001; Fig. 2C).

**Fig. 3:**
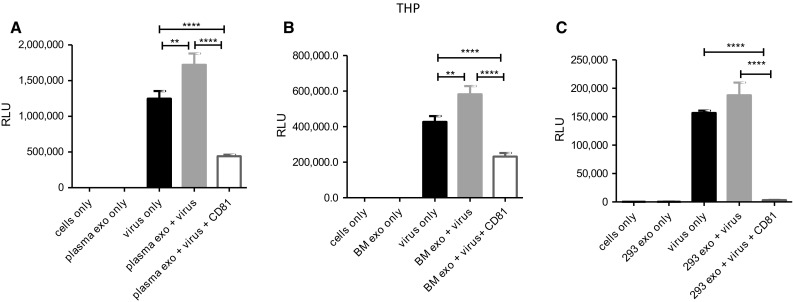
Exosomes significantly enhance HIV-1 entry into a human macrophage cell line. THP2574 cells (1x10^4^ cells/well) were classically differentiated into macrophages with 1 ng/ml phorbol 12-myristate 13-acetate) for 96 h. Virus was used in THP2574 cells at an MOI of 0.18. Virus entry into THP2574 cells was evaluated in the presence or absence of **(A)** plasma-derived exosomes, (plasma exo) **(B)** breast milk-derived exosomes, (BM exo) or **(C)** 293-derived exosomes (293 exo). Viral entry was also evaluated in the presence of exosomes and anti-CD81 antibody

In THP2574 macrophage-like cells virus alone entered the cells and yielded approximately 1.2x10^6^ RLU (Fig. 3A). Co-incubation of virus and plasma-derived exosomes increased the number of RLU significantly p=0.0018. Administration of plasma-derived exosomes, virus, and anti-CD81 led to a significant decrease in RLU compared to the administration of plasma-derived exosomes (p<0.0001) plus virus (Fig. 3A) or virus only (p<0.0001) (Fig. 3A). Similar trends were observed with exosome-mediated virus entry using breast milk-derived exosomes (p=0.0034; Fig. 3B). Next, anti-CD81 antibody was co-incubated with exosomes and virus then subsequently added to cells. CD81 exosome-mediated blockage of virus entry and gene expression further illustrated the specificity of CD81/exosome interactions in multiple human cell types (Figures 2 and 3).

### Breast milk-derived exosomes enhance HIV-1 entry and can be blocked using CD9 antibodies

**Fig. 4:**
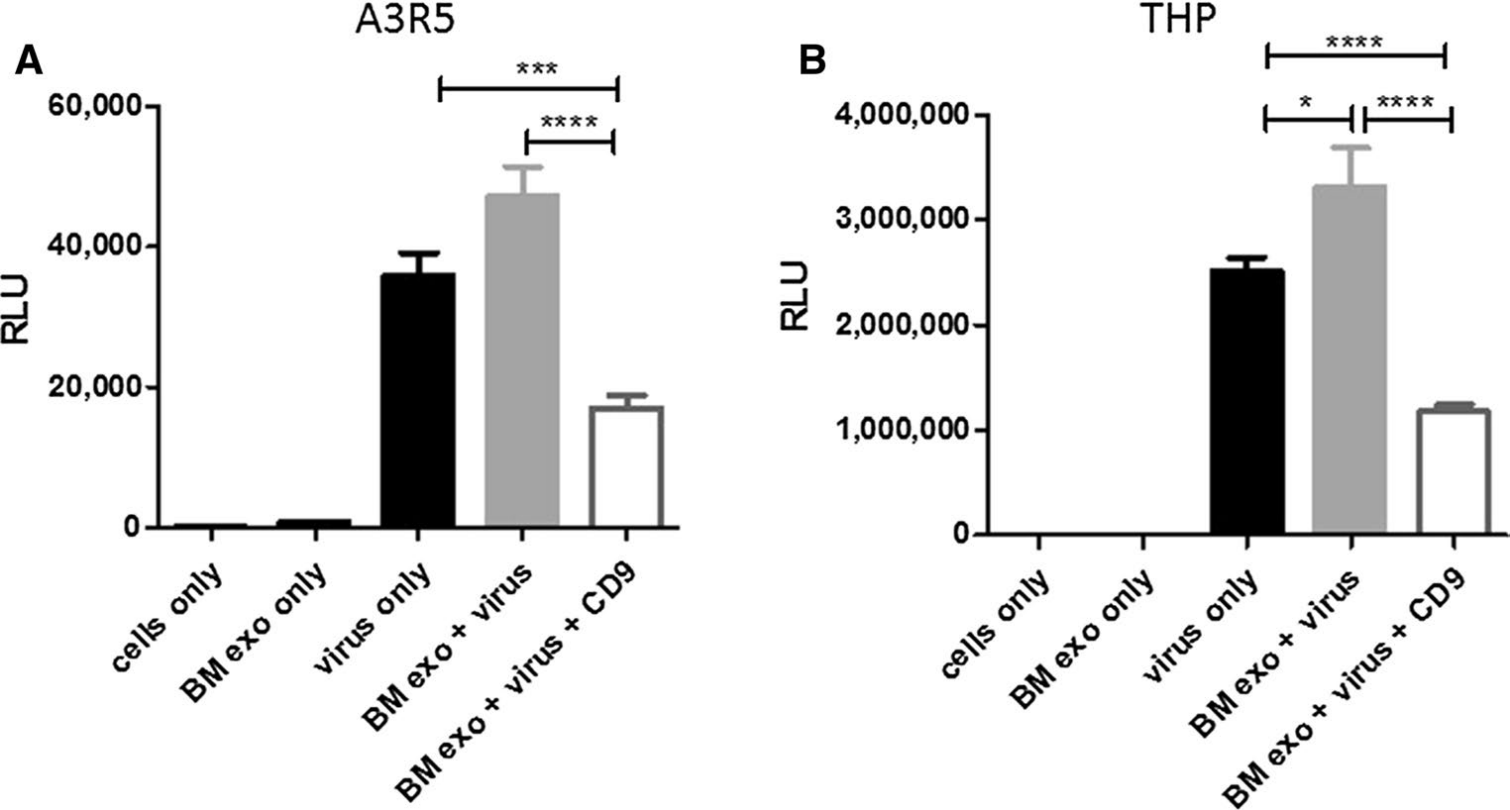
Breast milk-derived exosomes significantly enhance HIV-1 entry into human immune cell lines. Virus entry into **(A)** A3R5.7 cells or **(B)** THP2574 cells was evaluated in the presence or absence of breast milk-derived exosomes (BM exo). Viral entry was also evaluated in the presence of exosomes and anti-CD9 antibody

In order to evaluate the impact of CD9 on exosome-mediated HIV-1 entry, we performed several small-scale experiments identical to the virus and exosome blocking experiments shown in Figs. 2 and 3. For these studies we evaluated exosomes derived from breast milk. The addition of breast milk-derived exosomes with virus increased virus entry and subsequent gene expression in A3R5.7 cells (Fig. 4A); however, this exosome-mediated entry was significantly blocked following the addition of CD9 antibodies (p=0.0006) (Fig. 4A). When these experiments were performed in the recipient THP2574 macrophage like cell line, similar results were obtained (Fig. 4B). Breast milk-derived exosomes increased virus entry and gene expression and was subsequently blocked by the addition of CD9 antibody (p<0.0001).

## Discussion

Several biological processes involve tetraspanins such as membrane fusion, cell motility, protein trafficking and cell adhesion, to name a few [25]. Specifically, both CD9 and CD81 are thought to bind with the cytoskeleton via ezrin-radixin-moeisin (ERM) family proteins binding to actin [26]. CD9 and CD81 also have binding partners that are critical for cell adhesion, ICAM-1 and VCAM-1 [27]. Tetraspanins, in particular CD9 and CD81, are found in extracellular vesicles such as exosomes. Thus, it is very plausible that these molecules may be involved in exosome-cellular interactions.

It has been well documented that HIV-1 exploits the exosomal machinery, as reviewed by Madison and Okeoma [28]. We now know the importance of exosomal TIM-4 on viral entry in neural stem cells [8]. We have published that HIV-1 binds to TIM-4 in exosomes and this interaction is associated with enhanced cellular entry [9]. The role of exosomes in HIV-1 pathogenesis is complex and many proteins may be involved, including CD9 and CD81. Based on the current report and our previous studies, it appears all three proteins (TIM-4, CD9 and CD81) are involved. CD9 and CD81 are critical for cell membrane interaction/fusion. CD81 associates with CD4 and is critical for HIV-1 infection [29]. These processes may still be dependent on the specific exosomes involved in exosome-cellular entry.

The role of exosomes in HIV-1 pathogenesis is complex. Näslund and group demonstrated that exosomes from breast milk inhibit HIV-1 infection of dendritic cells and subsequent viral transfer to CD4^+^ T cells [30]. These findings are different from our findings herein, this may be attributed to the recipient cell types examined in our experiments, T lymphoblastoid cell line or the macrophage-like cell line. In this study, we evaluated exosomes derived from HEK 293 cells to gain some preliminary knowledge related to kidney cell-derived exosomes and their role in mediating HIV-1 entry. To our knowledge, this is the first study in this context. These finding might add to the understanding and further clinical advances of HIV-1 associated nephropathy (HIVAN) which is poorly understood [31].

A role for CD81 has been identified in HIV-1 budding, cell-to-cell spread and infectivity [1, 28, 29, 32–34]. It has previously been demonstrated that blocking CD9 and CD81 tetraspanins, which are commonly expressed on exosomes, resulted in significant decreases to exosomal uptake efficiency in dendritic cells [10]. These results are similar to the results we report here. Our current study was designed to evaluate exosome-mediated entry and not virus-antibody interaction. Based on our studies with TIM-4 in exosomes, HIV-1 binds to the exosome and proteins like CD9 and CD81 may then facilitate cellular entry. Research by Luo et al., (2015) revealed the existence of two different forms of exosomes: i) AChE+/CD81 low/TSG101 low exosomes and ii) AChE-/CD81 high/TSG101 high exosomes [35]. We demonstrate that various human-derived exosomes increase the entry of virus and subsequently that this can be blocked with the addition of CD81 and CD9 antibodies, respectively. Manipulating CD81 and CD9 binding to cells may be critical for exosomal entry. Therefore, blocking exosome-mediated uptake of HIV-1 may act as a therapeutic axis to reduce HIV-1 infections, particularly in latent HIV infected targets in immune privilege sites such as the brain.

## Abbreviations

HEK 293: Human embryonic kidney cells
TIM4: T cell immunoglobulin and mucin protein 4
NTA: Nanoparticle tracking analysis
HIV-1: Human Immunodeficiency virus-1
CD: cluster of differentiation
IMC: infectious molecular clone
LucR: *Renilla* luciferase
HAND: HIV-associated neurocognitive disorder
